# Therapeutic approach in an Alzheimer’s Disease rat model through GDNF overexpression by an astrocyte‐selective adeno‐associated virus

**DOI:** 10.1002/alz.089831

**Published:** 2025-01-03

**Authors:** Ana Abril Vidal Escobedo, Facundo Peralta, Paula Cecilia Reggiani, Joaquín Pardo

**Affiliations:** ^1^ National Council of Scientific and Technical Research (CONICET/UNLP), La Plata Argentina

## Abstract

**Background:**

Sporadic Alzheimer’s disease (sAD) is the most common form of dementia, characterized by a progressive decline in cognitive function and, cortical and hippocampal atrophy. Our objective is to develop neuroprotective therapies that promote the homeostatic and modulatory properties of astrocytes, and enhance their protective functions. Glial‐derived neurotrophic factor (GDNF) has emerged as a promising factor for its ability to promote neuronal survival and function. However, the therapeutic potential of GDNF in AD remains uncertain. Consequently, we explored it in the sAD model induced by intracerebroventricular (icv) streptozotocin (STZ) in rats.

**Method:**

We produced serotype 9 bicistronic adeno‐associated viruses (AAV), expressing GDNF and GFP (control), driven by the gfaABC1D promoter, targeting astrocytes expressing glial fibrillary acidic protein (GFAP). AAVs were characterized using RT‐qPCR and immunohistochemistry (IHC). For therapeutic approach, young male rats were divided into 4 groups: SHAM, STZ, GFP, and GDNF. On Experimental Day (ED) ‐28, animals received bilateral intrahippocampal injections of artificial cerebrospinal fluid (aCSF) (SHAM and STZ groups) or AAV‐GFP/GDNF (GFP and GDNF groups). On ED 0, animals received bilateral icv injections of aCSF (SHAM group) or STZ (3mg/kg) (STZ, GFP, and GDNF groups). Between ED +17/+26, behavioral tests were conducted to evaluate species‐typical behavior, object recognition memory, and spatial memory.

**Result:**

AAV‐GDNF and AAV‐GFP were generated, and the overexpression of transgenes was confirmed by RT‐qPCR and IHC. In the therapeutic approach, animals belonging to STZ and GFP groups showed significant deterioration in all assessed behaviors. GDNF overexpression prevented cognitive impairment in treated animals, as they showed no significant differences compared to the SHAM group in the analysed parameters. Additionally, AAV‐GDNF treatment promoted an increase in branching and length of processes in the analysed astrocytes

**Conclusion:**

We investigated an innovative therapeutic method specifically focused on hippocampal astrocytes. This allowed us to overexpress GDNF, generating protective actions that mitigated behavioral and cognitive alterations in our sAD animals. Furthermore, by acting on the brain’s astrocyte population, promoting an increase in their complexity, we could potentially have a positive effect on maintaining cerebral homeostasis.

